# Successful management of a ruptured cystic artery pseudoaneurysm with embolization and cholecystectomy: A case report

**DOI:** 10.1002/ccr3.9427

**Published:** 2024-09-15

**Authors:** Amirhossein Heidari, Yekta Ghane, Nazila Heidari, Amir Kasraianfard, Mahsa Kargar, Ali Mohammad Moradi

**Affiliations:** ^1^ Faculty of Medicine, Tehran Medical Sciences Islamic Azad University Tehran Iran; ^2^ School of Medicine Tehran University of Medical Sciences Tehran Iran; ^3^ School of Medicine Iran University of Medical Sciences Tehran Iran; ^4^ Liver Transplant Research Center Tehran University of Medical Sciences Tehran Iran; ^5^ Department of Pathology, Cancer Institute Tehran University of Medical Sciences Tehran Iran

**Keywords:** case report, cholecystectomy, embolization, gall bladder, pseudoaneurysm

## Abstract

**Key Clinical Message:**

Cystic artery pseudoaneurysm is a rare phenomenon associated with cholecystitis. We describe the successful management of angioembolisation and cholecystectomy.

**Abstract:**

Cystic artery pseudoaneurysm (CAP) is a rare but clinically significant condition with various etiological factors. Cholecystitis is a prominent cause, often leading to inflammation‐induced arterial wall erosion and pseudoaneurysm formation. CAP can present with a range of symptoms, including hemobilia, upper GI bleeding, and jaundice. Despite its rarity, CAP warrants attention in emergency care due to its potential for life‐threatening arterial bleeding. Timely diagnosis is crucial, with imaging techniques playing a key role. Depending on the clinical context, management options include endovascular embolization and surgical intervention. Due to the limited cases, standard protocols remain elusive. A 64‐year‐old woman presented with abdominal pain, anorexia, and weight loss, prompting an evaluation for possible gallbladder cancer. She experienced sudden abdominal pain and upper gastrointestinal bleeding (hematemesis). Laboratory findings revealed leukocytosis, anemia, and abnormal liver function tests. Imaging showed gallbladder wall thickening, luminal contraction, and a pseudoaneurysm in the cystic artery. The patient underwent angioembolization followed by cholecystectomy, confirming acute cholecystitis and CAP with thrombosis. This case underscores the importance of early recognition and appropriate management in CAP, particularly when accompanied by acute cholecystitis.

## 1 INTRODUCTION

Cystic artery pseudoaneurysm (CAP) is a rare condition associated with biliary procedures resulting in vessel wall injury. Most frequently, it is caused by operative trauma following biliary surgery, particularly laparoscopic cholecystectomy.^1^ The other common reasons include inflammation, such as cholecystitis, vasculitis, as well as abdominal trauma.^2^ In the case of cholecystitis, vasa vasorum thrombosis occurs due to visceral inflammation of the arterial wall that leads to a localized defect in the vessel wall.^3^, ^4^ Despite being an unusual complication of cholecystitis, CAP is essential to acknowledge in emergency care since it can result in life‐threatening conditions when arterial bleeding occurs.

Several complications are found to be associated with CAP, including hemobilia, anemia, biliary obstruction, hemoperitoneum, and hemodynamic shock.^5^ Biliary hemorrhage, known as hemobilia, is an uncommon but severe cause of gastrointestinal bleeding.^6^ Quincke's triad describes the classic presentation of hemobilia: jaundice, right upper quadrant abdominal pain, and upper gastrointestinal bleeding. Here, we reported a case of CAP concomitant with acute cholecystitis presented with hematemesis and managed by angioembolization and cholecystectomy.

## 2 CASE HISTORY

A 64‐year‐old woman was admitted to our center with chief complaints of first abdominal pain, and then anorexia, and weight loss from several months ago. The patient was first introduced to the evaluation for the possibility of gallbladder cancer at the hepatobiliary clinic of Imam Khomeini Hospital (Tehran, Iran). On admission day, the patient suddenly developed abdominal pain and upper gastrointestinal (GI) bleeding (hematemesis). Subsequently, she was directly hospitalized from our clinic to the intensive care unit (ICU) of our hospital with the evaluation of the upper GI series for resuscitation and diagnosis. She had a previous medical history of hypertension and hyperlipidemia with a drug history of a combination of ½ losartan 50 mg/hydrochlorothiazide 12.5 mg twice a day and atorvastatin 20 mg once daily.

In addition, no prior history of surgical procedures, cancer, or related‐positive familial records was reported. During admission, her blood pressure was 130/90 mmHg, pulse rate was 110 beats per minute, respiratory rate was 20 per minute, and body temperature was 37°C. Physical examination revealed generalized tenderness, especially shifting to the epigastric and right upper quadrant area. Pale conjunctiva was also observed. Moreover, the patient had a complete blood count report from 2 weeks ago with a hemoglobin (HBG) level of 12.4 g/dL (NR: 12.0–15.5 g/dL).

## 3 METHODS

Regarding the laboratory data test and complete blood count profile (CBC), a decline of HBG at 7.2 g/dL (NR: 12.0–15.5 g/dL) and elevation of leukocyte counts (11,400 μL) were observed (normal range [NR]: 4500–11,000 μL). In addition, the fasting blood sugar level was elevated to 190 mg/dL (NR: 70–99 mg/dL). Moreover, in the case of live function tests, a decline of albumin of 2.6 g/dL (NR: 3.5–5.0 g/dL) was observed; however, bilirubin total of 0.8 mg/dL (NL: 0.3–1.0 mg/dL) and bilirubin direct of 0.4 mg/dL (0.1–0.3 mg/dL). Hepatobiliary enzymes were also elevated as follows: aspartate aminotransferase (AST) of 54 U/L (NR: 10–40 U/L), alanine aminotransferase (ALT) of 120 U/L (NR: 7–56 U/L), alkaline phosphatase (ALP) of 772 U/L (NR: 35–115 U/L). Moreover, total Iron‐Binding Capacity (TIBC) and iron were in the lower margin of NR (TIBC: 230 μg/dL, NR: 250–450 μg/dL; iron: 52 μg/dL, NR: 50–170 μg/dL), and ferritin: >1000 ng/mL (NR: 12–150 ng/mL) were reported. Further, the prothrombin time (PT) was slightly increased at 13.3 s (NR: 10–13), the international normalized ratio (INR) level was 1.1 (NR: 0.8–1.1), and activated partial thromboplastin time (aPTT) was declined at 25 s (NR: 30–40). No remarkable changes were reported concerning other laboratory tests. (Normal electrolytes, urine culture, and blood culture: No growth of pathogens).

**FIGURE 1:**
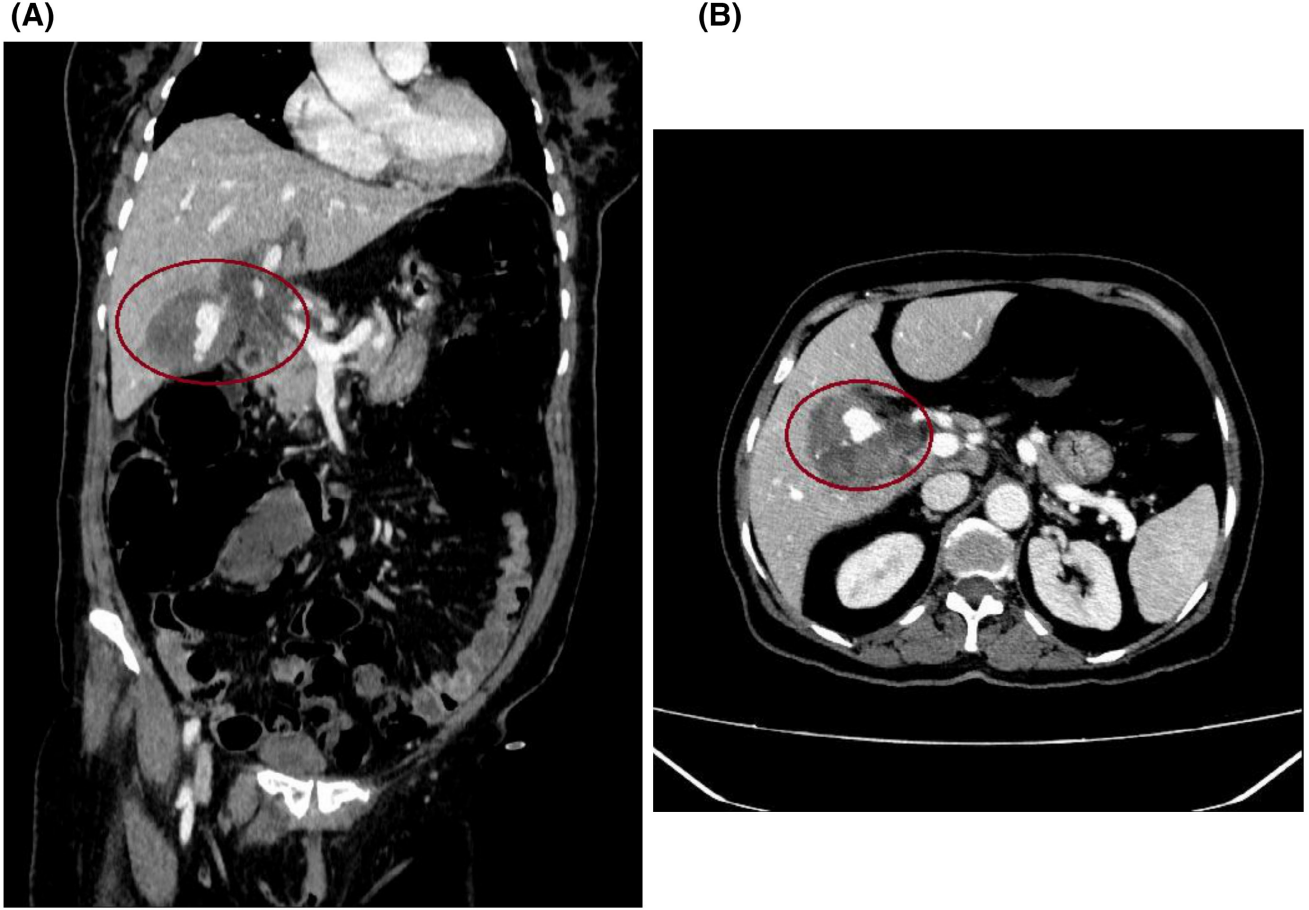
Contrast‐enhanced computed tomography scan of the abdomen. (A) In the coronal plane, an intra‐cholecystic pseudoaneurysm of the cystic artery with a hematoma around it was shown (red circle). (B) In the axial plane, a cystic artery pseudoaneurysm with surrounding hematoma was illustrated (red circle).

In the case of sonography, increasing the gallbladder's thickness with the mass inside it and the possibility of hematoma or bladder cancer was reported. In addition, upper GI endoscopy revealed coffee ground material in D1 and D2 in favor of hemobilia. The computed tomography (CT) scan with intravenous contrast demonstrated a number of considerable findings (Figure 1). The gallbladder showed an increased thickness of its wall, particularly pronounced in its medial wall, with a maximum diameter of 22 mm. Additionally, the gallbladder lumen appeared contracted and contained a stone measuring approximately 18 × 24 mm. Furthermore, calcifications were observed in certain areas of the gallbladder wall. The thickened wall of the gallbladder is consistent with a parietal hematoma, within which an elongated pseudoaneurysm measuring approximately 14 × 35 mm was visualized. This ruptured pseudoaneurysm is believed to originate from the cystic artery, which arises from the right hepatic artery. Another notable finding is the presence of hyperdense areas, suggestive of clot formation, in the middle and distal portions of the common bile duct (CBD). This clotting has led to the dilation of the internal bile ducts. Additionally, a hematoma was observed surrounding the gallbladder and ligamentum teres.

In the case of CAP management, angioembolization was performed to embolize the at‐risk ruptured pseudoaneurysm to stop and reduce further blood loss in subsequent cholecystectomy. The angioembolization details are as follows: a right femoral sheet was inserted under sterile conditions and local anesthesia. Guided by fluoroscopy and using a coaxial catheter system with a hydrophilic wire guide, microcatheter, and micro guidewire, angiography of the hepatic and cystic arteries was performed. A large pseudoaneurysm inside the gallbladder with leakage into the lumen of the gallbladder was observed. After consulting other radiology interventionists and considering end artery anatomy, the mentioned pseudoaneurysm was completely occluded using 500–700 units of polyvinyl alcohol (PVA) and five ccs of gel foam, and no more implementation was required, such as coil embolization. No complications were also observed during or after the procedure.

**FIGURE 2:**
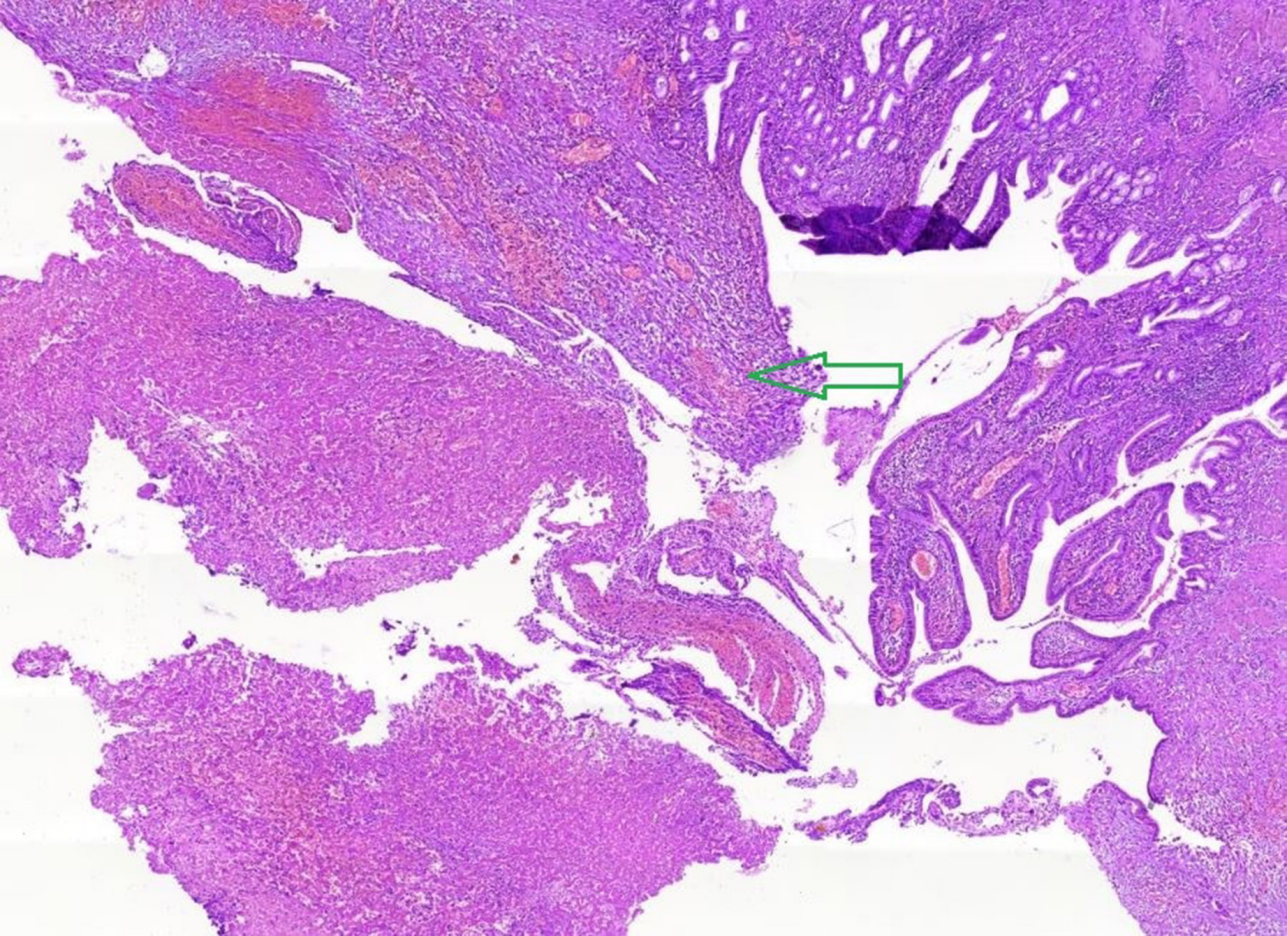
Hematoxylin and eosin (H&E) staining of the gallbladder sample demonstrated moderate infiltration along with ulcerated mucosa and the presence of polymorphonuclear neutrophils (PMNs) (green arrow) with 10x magnification.

**FIGURE 3:**
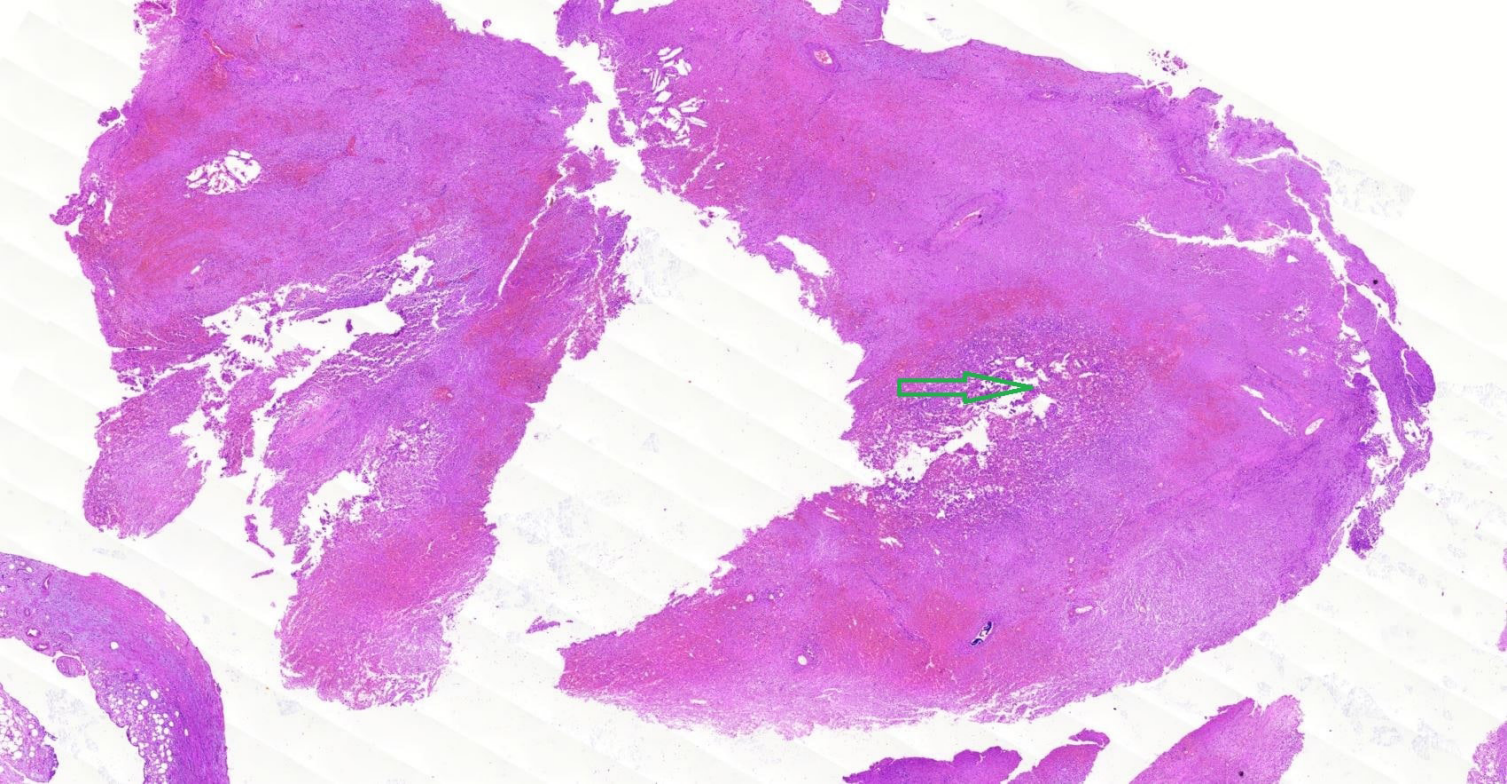
Hematoxylin and eosin (H&E) staining illustrated the evidence of organized and recanalized thrombus associated with cystic artery pseudoaneurysm (green arrow) with 10x magnification.

Concerning perioperative diagnosis of acute cholecystitis with the presence of ruptured CAP, laparoscopic surgery was conducted two days following embolization. Following general anesthesia, four laparoscopic ports were inserted, and the abdominal cavity was explored, revealing evidence of acute cholecystitis and omentum accumulation. A LigaSure™ laparoscopic instrument 5 mm connected to a Covidien electrosurgical generator (Valleylab™) was utilized to release omentum, duodenum, and colon adhesions to the gallbladder. Next, the CBD was evaluated, the suspicious region of CBD was minimally cut, and no evidence of any clot was observed. Further, the cystic duct was skeletonized, and in the proximal region of the cystic duct near the body of the gallbladder, one medium‐sized laparoscopic clip (purple clip) was ligated. After observation of the free flow of bile in the middle part of the cystic duct with partial cutting, the distal part of the cystic duct was ligated by two medium‐sized laparoscopic clips (purple clips), and the cystic duct was dissected. Nonetheless, the vascular anatomy of the hepatic hilum and CAP was subsequently studied but not identified. Additionally, due to a massive hematoma, gallbladder inflammation, and adherence to liver parenchyma, the laparoscopic procedure was converted to open surgery for a precise view of the surgery location and patient safety based on consulting other hepatobiliary surgeons during surgery. The gallbladder was attempted to be removed, but the posterior part was severely adhering to the liver parenchyma due to gangrenous changes. As follows, a large part of the gallbladder was dissected from the liver parenchyma, along with its pseudoaneurysm. In addition to ligating the cystic artery, the gallbladder and pseudoaneurysm cavity were completely resected. Finally, the body of CBD was completely palpated, and no evidence of any mass or clot was found. The CBD again washed through the cystic duct access, and the cystic duct was relegated with two medium‐sized laparoscopic clips (purple clips). Lastly, adequate hemostasis was achieved, and the surgical field was irrigated. After personnel determined that hemostasis had been completely provided and that gauze and instrument counts were accurate, a Nelaton drain was inserted in the CBD location to aid in bile drainage to the duodenum. All remaining mucosa of the gallbladder was also cauterized. The fascia was closed with nylon loop one while the skin was sutured with 3/0 nylon. After dressing at the surgical site, the patient was transferred to the recovery room in stable condition. Histopathological examination of the resected gallbladder specimen showed the phenomenon of acute cholecystitis (Figure 2) and thrombosis associated with CAP (Figure 3), confirming our final diagnosis.

## 4 RESULTS

Within the first day after admission, stabilization of the patient's clinical condition was performed. Considering the patient's HBG level of 7.2 g/dL, a comprehensive and immediate management plan was implemented prior to embolization. The patient also received two units of packed cells to elevate the HBG level and enhance oxygen‐carrying capacity. Additionally, two more units of packed cells were reserved for further transfusion if required. Continuous monitoring of the patient's vital signs, including heart rate, blood pressure, and oxygen saturation, was conducted to ensure hemodynamic stability and promptly address any signs of clinical deterioration.

Moreover, extensive laboratory assessments and paraclinical evaluations were performed to establish a definitive diagnosis and guide further management. Angioembolization and cholecystectomy were performed during 2 and 4 days after admission, respectively. Finally, 8 days after admission, the patients were discharged from our center. The patient responded to the procedures satisfactorily, with stable vital signs, good oral intake, regular bowel habits, and relief from abdominal pain and hematemesis. Laboratory tests performed postoperatively showed normal results. Following discharge from the surgical ward, the patient was given instructions regarding proper diet and medication use.

## 5 DISCUSSION

Regarding the low incidence of CAP, the clinical features have not been thoroughly investigated for a long time. In this paper, we describe a recent case of this rare disease to characterize it. However, the pathophysiology of CAP in cholecystitis is unclear; some etiologies seem to be associated with this condition. CAP is likely caused by inflammation, such as acute cholecystitis or even pancreatitis, leading to the development of pseudoaneurysms.^5^, ^7^ CAP formation can also be caused by biliary surgery, and recent reports suggest that CAP can be associated with other invasive liver and biliary procedures, in particular cholecystectomy.^8^, ^9^ The prior investigation reported that CAP symptoms could develop for up to 3 years after cholecystectomy.^10^ Additionally, some cases experienced the CAP in the context of idiopathic causes.^11^

Based on previous literature, cholecystitis is one of the most common causes of CAP formation among CAP cases.^12^ The widely endorsed understanding holds that inflammation results in erosion of the cystic arterial wall, causing adventitial damage and thrombosis, which eventually destroys the muscle and elastic structures of the intima and media, resulting in the formation of pseudoaneurysm with arterial extravasation.^11^, ^13^ Moreover, robust evidence indicated that gallstone presentation was found in 91%, gallbladder ruptures in 83%, and intraperitoneal in 18% of patients concomitant with cholecystitis.^8^ In our presented case, we hypothesize that the formation of a CAP can be attributed to regional inflammation associated with acute cholelithiasis. In addition, patient‐related conditions are likely to facilitate the formation, including atherosclerosis, hypertension, bleeding problems, diabetes, hypercholesterolemia, and vasculitis.^13^ As reported in our case, a positive previous history of hypertension could play a pivotal role in developing the CAP condition. Previous literature also suggested that the incidence of CAP was considerably higher among older adults. According to a recent study that analyzed 59 reported cases of CAP, approximately 55.9% of the individuals suffering from CAP were aged over 70 years.^12^ This result is in keeping with our case's age category.

On the first presentation, patients may experience nausea, vomiting, and vague abdominal pain during the early stages.^14^ In most cases, patients present with haemobilia or/and haemoperitoneum, biliary obstruction, anemia, or upper GI bleeding, like our case.^15^ The classic manifestation of haemobilia, referred to as Quincke's triad, is characterized by a constellation of symptoms.^6^ This triad can also be identified in cases involving CAP, where there is a variable incidence of upper abdominal pain (82.1%), GI hemorrhage (64.4%), and jaundice (58%).^12^ It is noteworthy that approximately 32% to 40% of patients present with the simultaneous occurrence of all three symptoms. Furthermore, as previously detailed, the laboratory examinations conducted on patients with CAP showed that elevated ALP levels were present in 90.6% of cases, hyperbilirubinemia in 92.1%, anemia in 80.5%, and leukocytosis in 75% of subjects.^12^ Considering these results, due to the hemorrhage from CAP into the gallbladder, our patient developed haemobilia, with subsequent biliary colic and obstructive jaundice, as marked by liver function tests (LFTs), anemia, and leukocytosis at the admission time.

Nevertheless, it is imperative to note that the clinical diagnosis of haemobilia is challenging upon presentation and requires a high grade of caution as well as utilizing imaging modalities as soon as possible.^2^ Abdominal ultrasound stands out as the most convenient and safest method for CAP in an acute context; however, it exhibits low sensitivity, which might undetect the CAP. Another choice is contrast‐enhanced CT scans, which usually identify CAP with more information and are available at most treatment centers.^2^ Color Doppler ultrasound and magnetic resonance imaging (MRI) can be performed as alternatives for patients who cannot receive intravenous contrast agents. It should be noticed that contrast CT/MRI with arterial phase is highly sensitive and specific over ultrasonography; conventional angiography is the gold standard due to its therapeutic potential.^1^

Considerably, a 50% mortality rate is estimated for CAP, primarily caused by hemorrhagic shock.^11^ Accordingly, the objective of managing CAP is to mitigate the risk of rupture and subsequent hemorrhage. Due to its infrequent incidence, a standardized protocol for managing CAP has yet to be established. As of yet, the most common method of controlling CAP, endovascular embolization, was demonstrated by previous studies to be successful for the majority of patients on their first attempt.^2^ If the endovascular approach fails, cholecystectomy surgery or vessel ligation is recommended.

## 6 CONCLUSION

In conclusion, taking into account the uncommon nature of this condition, the aetiologies listed do not establish a definitive cause‐and‐effect relationship, and it remains challenging to evaluate the comparative therapeutic efficacy of different management approaches. The most robust correlation that can be drawn pertains to the clinical presentation of CAP, as these presentations can be directly substantiated through gold‐standard confirmatory diagnostic imaging techniques and managed by proper procedures regarding patients' conditions and comorbidities. We presented a CAP case complaining of sudden abdominal pain and hematemesis. In our case, the laparoscopic approach failed to treat acute cholecystitis with gangrenous manifestations, and open surgery was performed for cholecystectomy following CAP angioembolization.

## AUTHOR CONTRIBUTIONS

**Amirhossein Heidari:** Data curation; investigation; validation; visualization; writing – original draft; writing – review and editing. **Yekta Ghane:** Data curation; investigation; validation; writing – original draft; writing – review and editing. **Nazila Heidari:** Data curation; investigation; validation; writing – original draft; writing – review and editing. **Amir Kasraianfard:** Investigation; methodology; validation; writing – review and editing. **Mahsa Kargar:** Data curation; visualization; writing – review and editing. **Ali Mohammad Moradi:** Conceptualization; methodology; project administration; supervision; validation; visualization; writing – review and editing.

## FUNDING INFORMATION

No funding supported this case report.

## CONFLICT OF INTEREST STATEMENT

The authors declared no conflict of interest.

## ETHICS STATEMENT

All procedures followed were in accordance with the ethical standards of the responsible committee on human experimentation (institutional and national) and with the Helsinki Declaration of 1975, as revised in 2000.

## CONSENT

Written informed consent was obtained from the patient to publish this report in accordance with the journal's patient consent policy.

## Supporting information


Data S1.

## Data Availability

Our data included personal patient data. Additional data are available from the corresponding author upon reasonable request.
